# Evaluation of Antidiabetic Activity of Oxadiazole Derivative in Rats

**DOI:** 10.1155/2023/1141554

**Published:** 2023-04-25

**Authors:** Adil Iqbal Qazi, Bashir Ahmad, Muhammad Umar Khayam Sahibzada, Fareeha Anwar, Ameer Khusro, Fahad A. Alhumaydhi, Amany Abdel-Rahman Mohamed, Gomaa Mostafa-Hedeab, Talha Bin Emran

**Affiliations:** ^1^Riphah Institute of Pharmaceutical Sciences, Riphah International University, Lahore, Punjab, Pakistan; ^2^Department of Pharmacy, The Sahara College Narowal, Narowal, Punjab, Pakistan; ^3^Centre for Research and Development, Department of Biotechnology, Hindustan College of Arts & Science, Padur, OMR, Chennai 603103, India; ^4^Department of Medical Laboratories, College of Applied Medical Sciences, Qassim University, Buraydah 52571, Saudi Arabia; ^5^Department of Forensic Medicine and Toxicology, Zagazig University, Zagazig 44511, Egypt; ^6^Pharmacology Department & Health Research Unit, Medical College, Jouf University, Saudi Arabia; ^7^Department of Pharmacy, BGC Trust University Bangladesh, Chittagong 4381, Bangladesh; ^8^Department of Pharmacy, Faculty of Allied Health Sciences, Daffodil International University, Dhaka 1207, Bangladesh

## Abstract

The oxadiazole ring has long been used for the treatment of several diseases. This study aimed to analyze the antihyperglycemic and antioxidant roles of the 1,3,4-oxadiazole derivative with its toxicity. Diabetes was induced through intraperitoneal administration of alloxan monohydrate at 150 mg/kg in rats. Glimepiride and acarbose were used as standards. Rats were divided into groups of normal control, disease control, standard, and diabetic rats (treated with 5, 10, and 15 mg/kg of 1,3,4-oxadiazole derivative). After 14 days of oral administration of 1,3,4-oxadiazole derivatives (5, 10, and 15 mg/kg) to the diabetic group, the blood glucose level, body weight, glycated hemoglobin (HbA1c), insulin level, antioxidant effect, and histopathology of the pancreas were performed. The toxicity was measured by estimating liver enzyme, renal function, lipid profile, antioxidative effect, and liver and kidney histopathological study. The blood glucose and body weight were measured before and after treatment. Alloxan significantly increased blood glucose levels, HbA1c, alanine transaminase, aspartate aminotransferase, urea, cholesterol, triglycerides, and creatinine. In contrast, body weight, insulin level, and antioxidant factors were reduced compared to the normal control group. Treatment with oxadiazole derivatives showed a significant reduction in blood glucose levels, HbA1c, alanine transaminase, aspartate aminotransferase, urea, cholesterol, triglycerides, and creatinine as compared to the disease control group. The 1,3,4-oxadiazole derivative significantly improved body weight, insulin level, and antioxidant factors compared to the disease control group. In conclusion, the oxadiazole derivative showed potential antidiabetic activity and indicated its potential as a therapeutic.

## 1. Introduction

Diabetes mellitus (DM) is often simply referred as a group of metabolic diseases in which a person has high blood sugar as well as altered fat, carbohydrate, and protein metabolism because either the body does not produce enough insulin (Type I) or cells do not respond to the insulin (Type II) [1]. This high blood sugar produces the classical symptoms of polyuria (frequent urination), polydipsia (increased thirst), and polyphagia (increased hunger) [2]. Type 1 diabetes occurs due to the selective destruction of beta cells of the pancreas [3]. The outcome of this destruction is the decreased production of insulin and its secretion from pancreatic cells [4]. Both environmental and genetic factors are involved in the pathogenesis of Type 2 DM. Among the diabetic subjects, about 75% of patients are affected by Type 2 DM. Thus, this type of diabetes is more prevalent than Type 1 diabetes [5].

Although the future cannot be predicted with certainty, it is surely possible that an extensive epidemic of resistance to antidiabetic drugs could potentially harm millions of people. Given that it takes more than 10 years to establish the efficacy and safety of the new compound, there is an urgent need to restock the antidiabetic agents. Only a few new antidiabetic compounds have received approval from the US Food and Drug Administration [6]. As we learn more about fundamental pathophysiological pathways, one of these is a lack of glucose stimulated insulin sensitivity as well as the prominence of lipotoxicity as a possible reason for the hepatic and muscular resistance to the effects of insulin on glucose metabolism [7].

**Figure 1:**
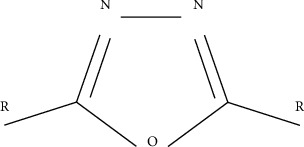
1,3,4-oxadiazole ring.

Literature survey reveals 1,3,4-oxadiazole derivatives (Figure 1), which belong to an important group of heterocyclic compounds, and it has been the subject of extensive study in the recent years. Numerous reports have highlighted their chemistry and uses. Diverse biological activities, such as antimycobacterial, antioxidant, antimicrobial, anti-inflammatory, analgesic, antipyretic, anticonvulsant, and antidiabetic, have been associated with the 1,3,4-oxadiazole derivatives [8–10].

1,3,4-oxadiazole derivative exerts its effects on diabetes by inhibiting the carbohydrate hydrolyzing enzymes, i.e., *α*-amylase and *α*-glucosidase, while preventing oxidative stress is deemed as a practicable strategy for regulating postprandial glucose levels in diabetic patients [11]. 1,3,4-oxadiazole derivative reduces hydrolytic activity and enzyme substrate complex to induce a slower release of the hydrolysed product, either by orthosteric inhibition or allosteric inhibitors which have been reported as *α*-glucosidase inhibitor in many molecular hybrid designs [12]. Conceivably, this can result in decreased systemic glucose concentration, thus alleviating postprandial hyperglycemia and its associated complications. The association between 1,3,4-oxidazole derivative and Nrf2 (nuclear factor E2-related factor or nuclear factor erythroid 2) enhances antioxidant enzyme activity, which suppresses free radical levels and promotes the pancreas to produce insulin and reduce blood glucose levels [13].

**Figure 2:**
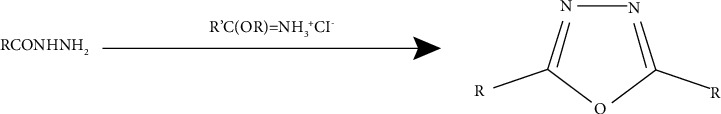
Preparation of 2,5-dialkyl(aryl)-1,3,4-oxadiazoles from acid hydrazide.

Oxadiazole occurs in four feasible interpretations of isomers, but 1,3,4-oxadiazole is broadly researched for different functions [14]. Oxadiazole rings were launched for various purposes in drug discovery development programs [15]. These were used as an essential member of the pharmacophore in some situations, thus leading to the ligand binding [16]. Various methods were reported in the literature for the synthesis of 1,3,4-oxadiazole and its derivatives [17]. The most widely applicable route to the synthesis of 1,3,4-oxadiazole and its 2,5-disubstituted derivatives is the thermal, acid, and base-catalyzed cyclisation of their corresponding carbonyl hydrazides. Preparation of 2,5-dialkyl(aryl)-1,3,4-oxadiazoles from acid hydrazide is shown in Figure 2 [18].

**Figure 3:**
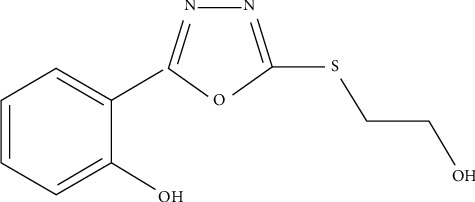
2-(5-[(2-hydroxyethyl)sulfanyl]-1,3,4-oxadiazole-2-yl)m phenol.

The ring of 1,3,4-oxadiazole is attached with the phenol and sulfanyl hydroxyethyl group (Figure 3). In view of the certain biological traits of 1,3,4-oxadiazole derivatives reported in the past, the present study was investigated to undertake further attempt towards the assessment of the antidiabetic role of 1,3,4-oxadiazole derivatives in rats.

## 2. Materials and Methods

### 2.1. Drugs and Chemicals

Alloxan monohydrate, hydrogen peroxide, sodium chloride, chloroform, sodium hydroxide, pyrogallol, potassium phosphate, sodium carbonate, glimepiride, acarbose, hydrochloric acid, folic acid, dextrose, trichloroacetic acid, and thiobarbituric acid (TBA) were purchased from Merck. Lidocaine was purchased from Pfizer company. DTNB (5,5-dithiobis-2-nitrobenzoic) acid was bought from the local market.

### 2.2. Equipment

Glucometer (Accu check), glucometer strip (Accu check), digital electronics weighing balance (Galvano scientific), vortex mixture (Vm-300), sonicator (Shakeel and Sons), spectrophotometer (Shimadzu UV-1601), centrifuge (Centurion scientific UK), homogenizer (Wise Tis HG-15A), refrigerator, digital water bath (HH-S4), binocular stereo microscope (YJ-T3c-China), and diagnostic kits (for the analysis of cholesterol, triglyceride, alanine transaminase or ALT, aspartate aminotransferase or AST, urea, and creatinine level) were used in this study.

### 2.3. Experimental Animals

Studies on albino Wister rats (weighing 150–200 g) of both sexes were performed. All rats were kept at room temperature (25 ± 1°C) under normal laboratory conditions for 12 h light-dark period with a humidity of 45–55%. A standard diet of pellets and water was fed before and during the experimental rats. All methods were issued by the Research Ethical Committee (REC/RIPS-LHR/011) of Riphah International University, Lahore, Pakistan.

### 2.4. Experimental Diabetes Induction

In this study, a single intraperitoneal injection (i.p.) of alloxan (150 mg/kg in 0.9% NaCl solution) was used to induce diabetes [19]. The intraperitoneal cavity of a 250 g rat could intake 2.5 mL of fluid [20]. The experimental animals were fasted for 18 h before injecting alloxan [21].

### 2.5. Study Design

Rats were divided into seven groups, and each group contained 6 animals. Treatment with the compound was started on the 3^rd^ day of alloxan treatment. Diabetic rats were known to have a blood glucose level greater than 180 mg/dL. All treatments were given orally once a day according to the body weight. The duration of the study was 14 days. Group I: control group with normal saline (5 mL/kg p.o.), Group II: diseased group with alloxan monohydrate (150 mg/kg i.p.), Group III: treated with glimepiride (4 mg/kg p.o.), Group IV: treated with acarbose (40 mg/kg p.o.), Group V: treated with 1,3,4-oxadiazole derivative (5 mg/kg p.o.), Group VI: treated with 1,3,4-oxadiazole derivative (10 mg/kg p.o.), and Group VII: treated with 1,3,4-oxadiazole derivative (15 mg/kg orally).

### 2.6. Antidiabetic Study

#### 2.6.1. Estimated Blood Glucose Levels

Blood glucose levels were determined using a glucometer before diabetes induction and during treatment time. The blood samples were collected on basal, zero, 5^th^, 10^th^, and 14^th^ day from the tail tip using a glass capillary tube. Basal blood glucose is the normal blood glucose content attained before inducing diabetes, while the day on which diabetes is completely induced in rats before starting the treatment is called zero-day [19].

#### 2.6.2. Glycated Haemoglobin Estimation

The level of HbA1c in blood represents the average blood glucose level over the last 2–3 months. When diabetes is not well regulated, blood glucose levels will also be elevated, which will cause higher levels of HbA1c. Glycosylated hemoglobin is measured by the Nycocard reader by glycosylated hemoglobin kits (Axis shield, Norway).

#### 2.6.3. Serum Insulin Level

All animals were sacrificed on the 15^th^ day of treatment after anaesthetizing with isoflurane (diluted with 2% oxygen), and their blood samples were taken from cardiac puncture. The blood was then centrifuged for 15 min at 2500 rpm to extract blood serum. The concentration of insulin in serum was calculated using a commercially available DSL-1600 insulin device (Diagnostic Systems Laboratories, Inc., USA). The values for insulin were expressed as *μ*U/mL.

#### 2.6.4. Weight Variation

The body weight of rats was examined at basal, zero, 5^th^, 10^th^, and 15^th^ days of treatment. The effect of 1,3,4-oxidiazole derivative on the body weight of rats was examined on the 5^th^, 10^th^, and 15^th^ day of the study.

### 2.7. Estimation of Biochemical Assays

#### 2.7.1. Preparation of the Tissue Sample

To prepare tissue homogenate, the pancreas was taken from sacrificed animals. All the tissues (10% w/v) were washed with cold normal saline and then dissolved in 0.1 M of phosphate buffer (pH 7.4).

#### 2.7.2. Estimation of Protein

Tissues were adequately diluted with phosphate buffer for protein estimation, and 5 mL of copper sulfate reagent comprising 1% Na_2_CO_3_, 2% sodium potassium tartrate, and 1% CuSO_4_ was added. The mixture containing 0.5 mL of Folin–Ciocalteau phenol reagent was incubated for 10 min. The sample was incubated for 30 min before reading the absorbance at 620 nm [22].

The protein level was determined using the following equation:

(1) $$\begin{array}{c} Y=0.00007571x+0.00004762\text{.} \end{array}$$

#### 2.7.3. Determining CAT Activity

Phosphate buffer (1.95 mL; pH 7.0; and 50 mM) and 1 mL of 30 mM of hydrogen peroxide (H_2_O_2_) were added into the supernatant (0.05 mL). A change in the absorbance value was spectrophotometrically reported at 240 nm every 5 sec. The CAT activity was expressed as units per milligram of protein as compared to the standard [23].

(2) $$\begin{array}{c} CAT\;activity=\frac{\delta OD}{E}\times Vol.of\;the\;sample\left(mL\right)\times mg\;of\;protein\text{,} \end{array}$$

where *δ* OD is the change in absorbance per min and *E* is extinction coefficient (0.071 mmol·cm^−^1) of H_2_O_2_ [24].

#### 2.7.4. MDA Determination

The rates of MDA, an indicator of lipid peroxidation, were calculated using the double heating method. The procedure is based on spectrometric measurement of violet colour produced by the TBA reaction. For this purpose, 2.5 mL of TCA solution was added to each centrifuge tube containing 0.5 mL of the supernatant of the tissue preparation. Tubes were incubated in a boiling water bath for 15 min. Upon cooling to ambient temperature, the tubes were centrifuged for 10 min and 2 mL of each supernatant sample was mixed to 1 mL of TBA solution. Each tube was then placed for 15 min in a boiling bath. The absorbance was measured at 532 nm [25].

(3) $$\begin{array}{c} Concentration\;of\;MDA={Absorbance}_{532}\times 100\times \frac{Vt}{\left(1.56\times 10^{5}\right)}\times WT\times VU\text{,} \end{array}$$

where *VT* is total mixture volume (4 mL), 1.56 × 10^5^ is the coefficient of molar extinction, *WT* is the weight of the dissected brain (1 g), and *VU* is volume of aliquot (1 mL) [26].

#### 2.7.5. SOD Testing

Each reaction mixture contained 2.8 mL of potassium phosphate buffer, 0.1 mL of homogeneous tissue, and 0.1 mL of pyrogallol solution. Increased absorbance at 325 nm was spectrophotometrically reported at 30 sec intervals for 5 min [27].

#### 2.7.6. Estimation of GSH

For the calculation of reduced glutathione, 1 mL of homogenous tissue with 1 mL of 10% TCA was precipitated. Then, 4 mL of phosphate solution and 0.5 mL of DTNB reagent were applied to the supernatant aliquot, and the absorbance was read at 412 nm.

(4) $$\begin{array}{c} GSH\;level=Y-\frac{0.00314}{0.0314}\times \frac{DF}{BT}\times VU\text{,} \end{array}$$

where *Y* is the absorbance at 412 nm of tissue homogenate, *DF* is the dilution factor (1), *BT* is brain homogeneous tissue (1 mL), and *VU* is volume of aliquot (1 mL).

### 2.8. Histological Studies

The pancreas was blotted free of mucus from laboratory rats, washed in normal saline, and preserved in 10% of formaldehyde for 24 h, followed by implanting in paraffin. The kidney and liver were collected after dissection and fixed in the blocks of paraffin wax, followed by storing in 10% formalin solution. The pancreas, kidney, and liver were stained with hematoxylin-eosin for histopathological study.

### 2.9. Toxicological Study

The toxicological study of 1,3,4-oxadiazole derivative was observed by contrasting the degree of different parameters of biochemistry between the test group, control group, disease group, and normal group. The method of collecting blood and separating serum from blood has already been described earlier. Preparation of the supernatant from tissue is also described.

### 2.10. Biochemical Metabolic Parameters

Liver function tests viz. ALT and AST were analyzed by Crescent Diagnostics CZ 902L and Crescent Diagnostic CZ 904L kit, respectively. The kidney function test, i.e., the urea level was measured by Crescent Diagnostic CS612 kit, and the creatinine level was measured by Crescent Diagnostic CS604 kit. Triglyceride and cholesterol were analyzed by Crescent Diagnostic CS611 and Crescent Diagnostic CS603, respectively.

### 2.11. Antioxidant Activity

Superoxide dismutase was calculated according to the method of Kim et al. [27]. GSH and MDA activities were analysed by Bhangale and Acharya [26]. CAT was measured by Kaur [24], and protein content was estimated by Lowry et al. [22].

#### 2.11.1. Histological Studies

The kidney and liver were collected and fixed in the blocks of paraffin wax, followed by saving in 10% formalin solution. The kidney and liver were stained with hematoxylin-eosin for histopathological observations.

### 2.12. Statistical Analysis

Results were presented as the mean ± SEM (standard error mean). For graphical interpretation, GraphPad prism was applied. The test of two-way ANOVA (analysis of variance) was applied. The results were intimated as moderately significant (*P* < 0.05 and ^*∗∗*^*P* < 0.01) and as highly significant (^*∗∗∗*^*P* < 0.001).

## 3. Results

### 3.1. Antidiabetic Activity

#### 3.1.1. 1,3,4-Oxadiazole Derivative Effect on the Blood Glucose Level

**Figure 4:**
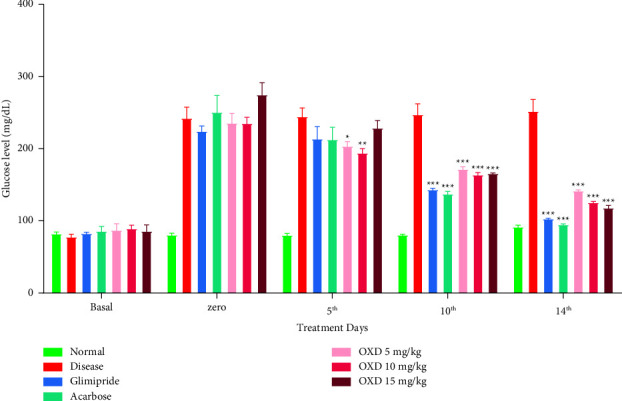
Effect of 1,3,4-oxadiazole derivative, glimepiride, and acarbose on the blood glucose level. Alloxanized rats were given either vehicle (carboxy methylcellulose; 1 mg/kg), 1,3,4-oxadiazole derivative (5, 10, and 15 mg/kg), glimepiride (4 mg/kg), or acarbose (40 mg/kg) orally once daily from day 1^st^ to 14^th^ day of treatment. The blood glucose levels were measured before alloxan (basal) and on zero (72 h after alloxan), 5^th^, 10^th^, and 14^th^ day of various treatments. The data represent mean ± SEM (*n* = 6). ^*∗∗∗*^*P* < 0.001, ^*∗∗*^*P* < 0.01, ^*∗*^*P* < 0.05 represents significantly different values as compared to the disease control, i.e., alloxanized rats. Note: OXD: 1,3,4-oxadiazole derivative.

1,3,4-oxadiazole derivative did not show a dose-dependent reduction in the glucose level, i.e., the degree of response was the same at all dose levels, indicating that maximum response can be achieved at the lowest dose, i.e., 5 mg/kg (Figure 4). However, the glucose reduction effect of all 1,3,4-oxadiazole derivative groups was found duration-dependent, indicating that a maximum effect was observed after the 14^th^ day of treatment. The blood glucose level of 1,3,4-oxadiazole derivative at 5, 10, and 15 mg/kg doses was reduced from 236 ± 14.7, 235 ± 9.9, 275 ± 17.9 mg/dL to 142 ± 2.8, 125 ± 3.2, 118 ± 4.9 mg/dL, respectively, from 0 to the 14^th^ day of treatment.

### 3.2. Treatment on Body Weight

**Figure 5:**
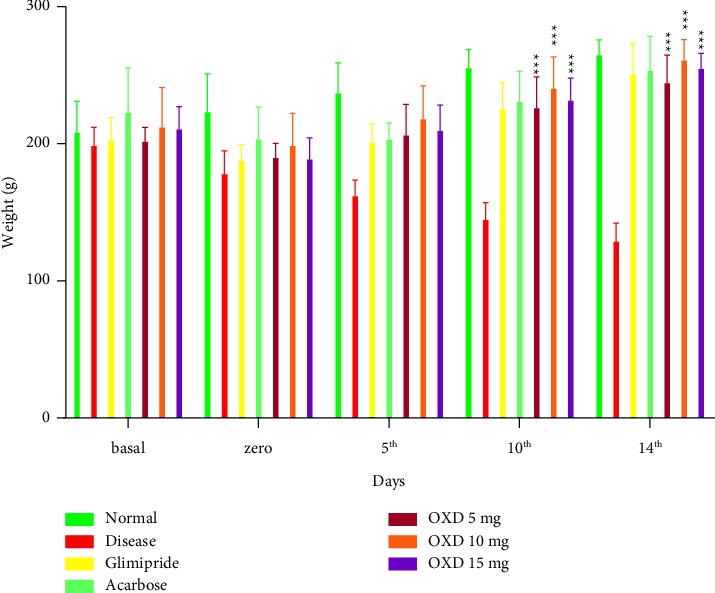
Body weight on various days of treatments. Each group (*n* = 6) represents mean ± SEM. ^*∗∗∗*^*P* < 0.001 represents significantly different values as compared to disease control. Note: OXD: 1,3,4-oxadiazole derivative.

Oral administration of 1,3,4-oxadiazole derivative at 5, 10, and 15 mg/kg to diabetic rats significantly (*P* < 0.001) increased the body weight (Figure 5).

### 3.3. Effect of 1,3,4-Oxadiazole Derivative on Glycated Hemoglobin (HbA1c) and Insulin Levels

After 14 days of treatment with 5, 10, and 15 mg/kg of 1,3,4-oxadiazole derivative, it resulted in significant (*P* < 0.001) attenuation of elevated HbA1c levels and was estimated as 0.56 ± 0.4, 0.44 ± 0.2, and 0.39 ± 0.3%, respectively, in contrast to the normal control group (0.37 ± 0.4%). The 1,3,4-oxadiazole derivative produced a dose-dependent reduction in the HbA1c level (Table 1). The multiple doses (5, 10, and 15 mg/kg) of 1,3,4-oxadiazole derivative also showed a dose-dependent increase in insulin levels as compared to the disease control group.

### 3.4. Effect of 1,3,4-Oxadiazole Derivative at Different Dose Levels on Biochemical Assays

The level of SOD was increased to 53.5 ± 0.5 *μ*g/mg in the diabetic group treated with 15 mg/kg of oxadiazole derivative in contrast to the disease control group (35.5 ± 2.5 *μ*g/mg). The CAT level was significantly (*P* < 0.05) increased to 123 ± 1.0 *μ*gmole/min/mg at a dose level of 15 mg/kg as compared to the disease control group which was recorded as 96 ± 0.5 *μ*gmole/min/mg. 1,3,4-oxadiazole derivative at a dose level of 15 mg/kg showed a significant (*P* < 0.05) increase in the level of the GSH level as compared to the disease control group. Administration of 1,3,4-oxadiazole derivative at 15 mg/kg to the diabetic rats reduced the level of MDA to 0.370 ± 0.005 *μ*mole/mg, which was a nonsignificant reduction as compared to the disease control group (0.390 ± 0.010 *μ*mole/mg). 1,3,4-oxadiazole derivative at a dose of 5, 10, and 15 mg/kg showed an increase in the protein level which was significantly different (*P* < 0.05, *P* < 0.01) in the disease control group **(**Table 2**)**.

### 3.5. Histopathological Study of Pancreas

**Figure 6:**
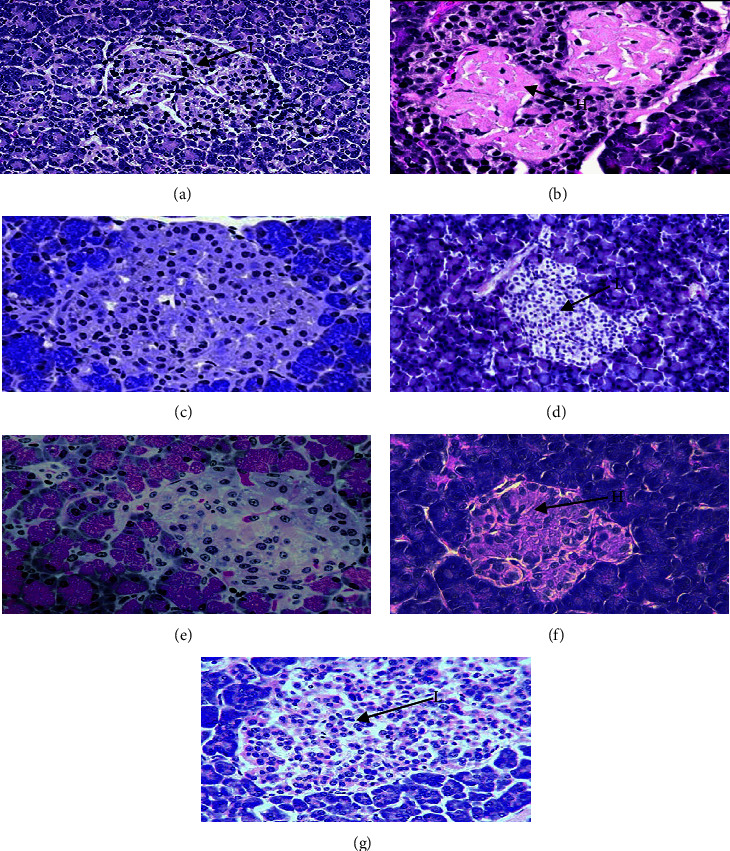
Microscopic image of rat pancreatic islets. (a) Normal pancreatic cells, (b) disease showing extensive damage to the islet of Langerhans and hemorrhagic (H) condition, (c) treatment with 5 mg/kg of 1,3,4-oxadiazole derivative showed hemorrhagic cells, (d) treatment with 10 mg/kg of 1,3,4-oxadiazole derivative showed vacuolization of *β* cells of islets of Langerhans, hemorrhage (H), (e) treatment with 15 mg/kg of 1,3,4-oxadiazole derivative showed hypertrophy and vacuolization of *β* cells of islets of Langerhans, (f) treatment with glimepiride showed little recovery of cells but still hemorrhage (H), and (g) treatment with acarbose showed hemorrhagic condition in *β* cells.

Histology of the pancreas demonstrated normal acini and cellularity in the normal control group. In diseased animals, extensive damage to islet of Langerhans and hemorrhagic condition can be seen clearly in Figure 6. On the other hand, 15 mg/kg of 1,3,4-oxadiazole derivative showed hypertrophy and vacuolations of *β* cells of Langerhans.

### 3.6. Toxicity Study

#### 3.6.1. 1,3,4-Oxadiazole Derivative Effect on Liver Enzymes

**Figure 7:**
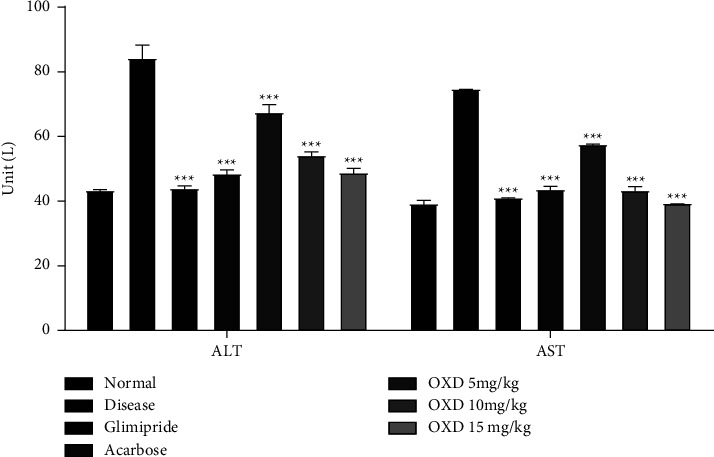
Effect of 1,3,4-oxadiazole derivative (5, 10, and 15 mg/kg), glimepiride (4 mg/kg), and acarbose (40 mg/kg) on ALT and AST levels in alloxanized rats. Each group (*n* = 6) represents mean ± SEM. ^*∗∗∗*^*P* < 0.001 represents significantly different values as compared to the disease group. Note: OXD: 1,3,4-oxadiazole derivative.

A statistically significant (*P* < 0.001) reduction in ALT level (48.95 ± 1.75 unit/L) was observed after 14 days administration of 15 mg/kg of 1,3,4-oxadiazole derivative with respect to the disease control (84.41 ± 4.38 unit/L). Similar results were observed in AST level (39.50 ± 0.50 unit/L) with administration of 15 mg/kg of 1,3,4-oxadiazole derivative as compared to the disease control group (74.85 ± 0.25 unit/L) (Figure 7).

#### 3.6.2. Effect of 1,3,4-Oxadiazole Derivative on Cholesterol, Triglyceride, and Urea Level

**Figure 8:**
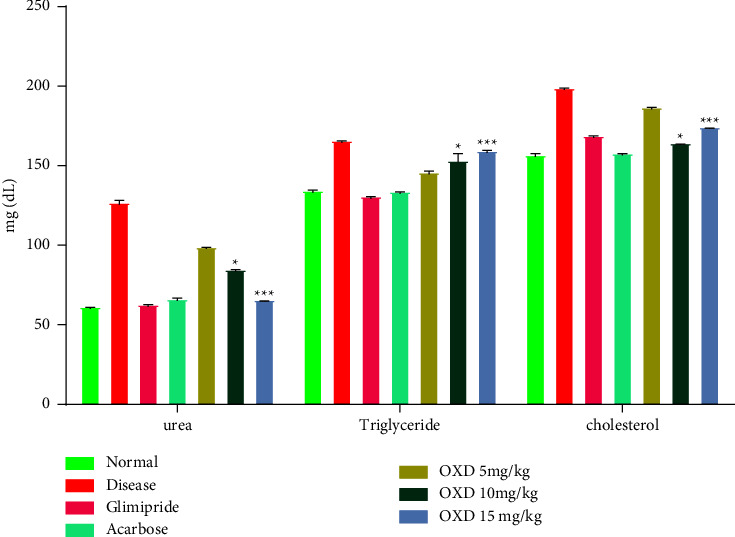
Effect of 1,3,4-oxadiazole derivative, glimepiride, and acarbose on urea, triglyceride, and the cholesterol level. Data are presented as mean ± SEM (*n* = 6). ^*∗∗∗*^*P* < 0.001 and ^*∗*^*P* < 0.05 represent significantly different values as compared to the disease group. Note: OXD: 1,3,4-oxadiazole derivative.

1,3,4-oxadiazole derivative showed the ability to significantly reduce the cholesterol level. After 14 days of treatment, it was found that at a dose level of 15 mg/kg, the cholesterol level was decreased (164 ± 0.5 mg/dL), which was significantly (*P* < 0.001) different as compared to the disease control group (199 ± 1.0 mg/dL). The administration of 1,3,4-oxadiazole derivative at the dose level of 15 mg/kg for 14 days significantly (*P* < 0.001) reduced the triglyceride level (131 ± 1.0 mg/dL) as compared to the disease control group (166 ± 1.0 mg/dL). Similarly, a significant (*P* < 0.001) reduction in the urea level was observed in the presence of 15 mg/kg of an 1,3,4-oxadiazole derivative (65.9 ± 0.4 mg/dL) as compared to the disease control group (127 ± 2.4 mg/dL) (Figure 8).

#### 3.6.3. Effect of Treatment on the Creatinine Level

**Figure 9:**
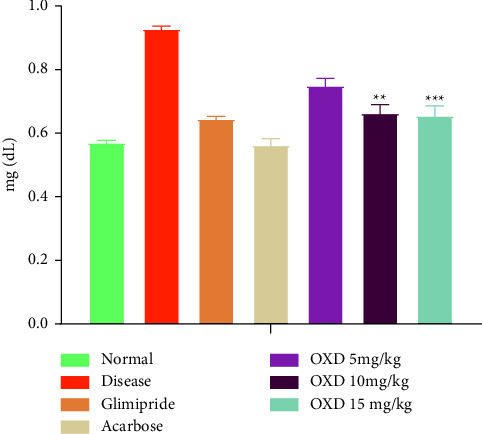
Effect of oxadiazole derivative, glimepiride, and acarbose on the creatinine level. Data are presented as mean ± SEM (*n* = 6). ^*∗∗∗*^*P* < 0.001 and ^*∗∗*^*P* < 0.01 represent significantly different values as compared to the disease control group. Note: OXD: 1,3,4-oxadiazole derivative.

Figure 9 illustrates the treatment outcomes of different doses of 1,3,4-oxadiazole derivative, alloxan, glimepiride, and acarbose on the creatinine level of test animals. The diseased control group exhibited high creatinine levels when compared to the normal control. The results of the study exhibited that treatment with 1,3,4-oxadiazole derivative at a dose level of 5, 10, and 15 mg/kg, glimepiride, and acarbose reduced the level of creatinine as compared to the disease control animals.

### 3.7. Antioxidant Activity of 1,3,4-Oxadiazole Derivative on Oxidative Biomarkers in Liver and Kidney Tissue

Table 3 illustrates the level of antioxidant components. Treatment of the diabetic rats with 15 mg/kg of 1,3,4-oxadiazole derivative decreased MDA levels to 0.901 ± 0.005 *μ*mole/mg as compared to the disease control group which was found as 1.300 ± 0.009 *μ*mole/mg in the liver. In addition, SOD and GSH activities at a dose level of 10 and 15 mg/kg of 1,3,4-oxadiazole derivative were significantly (*P* < 0.05) increased as compared to the disease control group in the liver. Similarly, the CAT activity in hepatic tissue was significantly (*P* < 0.01) increased by adding 1,3,4-oxadiazole derivatives at 15 mg/kg (0.891 ± 0.005 *μ*gmole/min/mg) as compared to the corresponding disease control group (0.450 ± 0.005 *μ*gmole/min/mg). Similarly, 15 mg/kg of 1,3,4-oxadiazole derivative significantly (*P* < 0.05) increased the protein level (454 ± 0.5 *μ*g/mg) as compared to the disease control group (341 ± 0.1 *μ*g/mg). However, in the kidney, the treatment with 1,3,4-oxadiazole derivative at a dose level of 15 mg/kg significantly increased (12.12 ± 0.52 *μ*g/mg) the level of reduced GSH on the 15^th^ day as compared to the disease control group (4.57 ± 1.4 *μ*g/mg). Treatment with 10 and 15 mg/kg of 1,3,4-oxadiazole derivative significantly (*P* < 0.05) elevated the level of CAT as compared to the disease control group in the kidney. Treatment with 1,3,4-oxadiazole derivative at a dose level of 10 and 15 mg/kg showed a significant (*P* < 0.01) increase in SOD activity (48.75 ± 0.54 *μ*g/mg and 48.90 ± 0.10 *μ*g/mg) as compared to the disease control group (44.05 ± 0.54 *μ*g/mg) in the kidney. In the case of protein level, only 15 mg/kg of 1,3,4-oxadiazole derivative showed a significant (*P* < 0.05) increase in the protein level in the kidney. Treatment with 1,3,4-oxadiazole derivative at a dose level of 5 and 10 mg/kg showed a significant (*P* < 0.01) reduction in MDA level as compared to the disease control group.

### 3.8. Histopathological Study of the Liver

**Figure 10:**
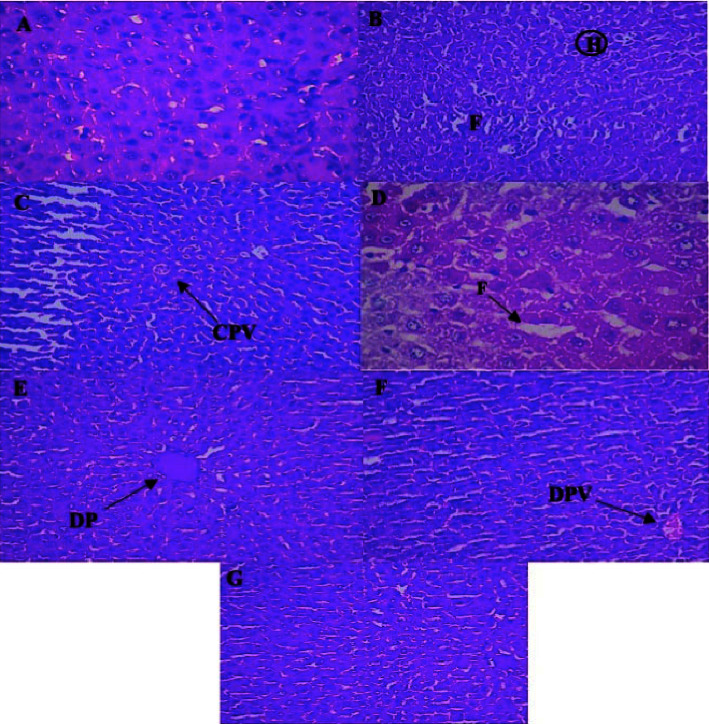
Histopathology of rat hepatic cells. (A) Normal hepatic cells showing preserved cytoplasm and nucleus, (B) disease rat showed area of hemorrhagic [H] and fatty change [F], (C) treatment with 5 mg/kg of oxadiazole derivative showed hepatic restoration with the congested portal vein [CPV], (D) treatment with 10 mg/kg of 1,3,4-oxadiazole derivative showed fat build up in the liver cells, (E) treatment with 15 mg/kg of 1,3,4-oxadiazole derivative showed restoration of normal hepatic architecture and dilation of the portal [DP] vein, (F) treatment with glimepiride showed recovery of hepatic cell with the dilation of portal vein [DPV], and (G) treatment with acarbose showed DPV with normal hepatic cells.

Hepatic microscopic examination of the normal group showed healthy liver cells with a well-preserved cytoplasm, nucleus, core nuclei, and major vein (Figure 10). The disease group displayed a necrotic region where blood drained from a ruptured blood vessel and obvious fatty impairment. The main vein detrimentally clogged up. Focal hemorrhage areas were also established. The fat change was evident. Kupfer cell activation and dilation of the central vein can be seen in the liver treated with 15 mg/kg of 1,3,4-oxadiazole derivative.

### 3.9. Histological Study of the Kidney

**Figure 11:**
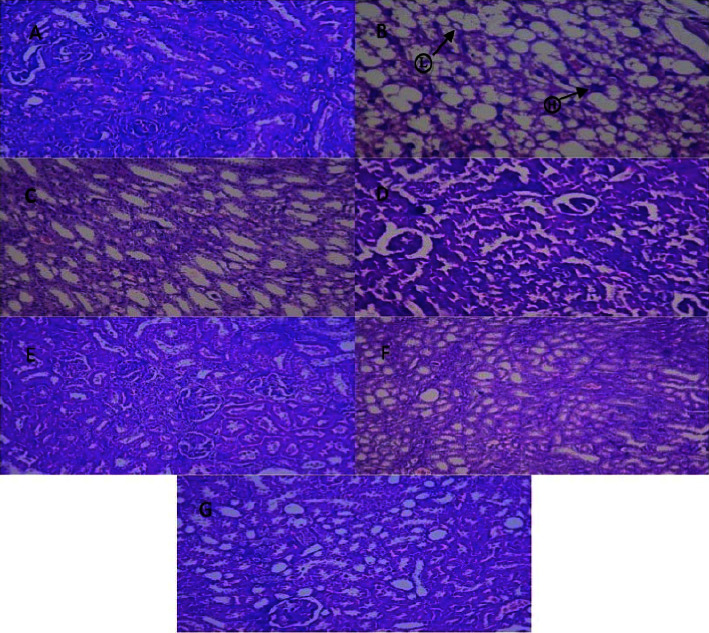
Microscopic view of renal cells. (A) Normal histology, normal glomeruli, and Bowman's capsule, (B) disease rate showed glomerular lipidosis [L], cellular proliferation with fibrosis, and some congestion in Bowman [H], (C) treatment with 5 mg/kg of 1,3,4-oxadiazole derivative showed rejuvenated tubules with hemorrhagic bowman, (D) treatment with 10 mg/kg of 1,3,4-oxadiazole derivative showed hemorrhagic Bowman with congestion of tubules, (E) treatment with 15 mg/kg of 1,3,4-oxadiazole derivative showed well-rejuvenated tubules with the glomerulus with no congestion and haemorrhage, (F) treatment with glimepiride showed hemorrhagic Bowman and degeneration of tubules, and (G) treatment with acarbose showed moderate haemorrhages in glomeruli.

In normal animals, the histology of the glomeruli and the Bowman capsule is appropriate. When lipid is deposited in the glomeruli of diseased rats, the basement membrane of the arterioles of the glomeruli becomes slightly thicker. This condition is known as glomerular lipidosis, and it includes fibrosis and cellular proliferation in the mesangial as well as congestion in the Bowman's as shown in Figure 11. After administration of 15 mg/kg of 1,3,4-oxadiazole derivative, the glomerulus and tubules were well rejuvenated and entirely free of congestion.

## 4. Discussion

Diabetes mellitus is the condition of hyperglycemia and derangement in carbohydrates, proteins, and fats due to the lack of insulin [28]. Worldwide estimates suggest that around 300 million people will get diabetes in 2025, and the global cost for the treatment of diabetes will reach US$1 trillion [29]. Alloxan is reduced to form diluric acid by binding with the SH sugar-binding site of glucokinase, an enzyme which is essential for glucose-induced insulin secretion causing hyperglycemia [30]. In this study, the administration of 1,3,4-oxadiazole derivative at a dose level of 5, 10, and 15 mg/kg for 14 days led to decrease in blood glucose levels, relative to the group of disease control. Alloxan-induced diabetes is distinguished by a massive weight loss due to the muscle loss and tissue protein catabolism, as seen at day zero [31]. Treatment with the 1,3,4-oxadiazole derivative, however, represented a significant gain in body weight as compared to the disease control group. Glycated hemoglobin is an extensively accepted parameter for the identification of plasma glycemic levels in a diabetic patient [32]. Protein glycation involves a series of complex reactions that occur between monosaccharides (glucose and fructose) and amino acids or proteins, producing an unstable Schiff base and then forming Amadori products such as fructosamine [33]. Alloxan has two distinct pathological effects, it selectively inhibits glucose induced insulin secretion through specific inhibition of glucokinase, the glucose sensor of the beta cell, and it causes a state of insulin-dependent diabetes through its ability to induce ROS formation. ROS causes the selective necrosis of beta cells leading to the increase in protein glycation, three days just after alloxan injection [34]. These two effects can be assigned to the specific chemical properties of alloxan, the common denominator being selective cellular uptake and accumulation of alloxan by the beta cell [35]. In this investigation, diabetic rats showed higher levels of Amadori product, suggesting their impaired glycemic control [36]. The dose level at 5, 10, and 15 mg/kg of 1,3,4-oxadiazole derivative showed a concentration-dependent reduction in Amadori product within 14 days, by activating the glucokinase enzyme and reduction in ROS production [37].

Insulin is a hormone made by the pancreas that binds to the glycoprotein receptor on the cell membrane of the cell and stimulates the transportation of glucose across the cell membrane by glucose transporter GLUT4 [38]. In diabetic rats treated with 1,3,4-oxadiazole derivative as compared to the diseased, an increase in plasma insulin levels was observed. This could be due to the potential of insulin sensitivity on the cell membrane [39]. Improving insulin sensitivity is the ultimate physiological effect of oxadiazole derivative in improving the homeostasis of glucose [40].

Diluric acid is oxidized to alloxan to generate the superoxide radical [41]. The SOD plays an important role in the metabolism of oxygen by reducing superoxide anion free radical (O_2_^−^) to nonreactive H_2_O_2_ and molecular oxygen [42]. At a dose level of 15 mg/kg, 1,3,4-oxadiazole derivative showed a significant (*P* < 0.001) rise in the SOD level when compared to the diabetic group. Catalase performs its activity to convert H_2_O_2_ into water and O_2_ and protect the cell from oxidative stress [43]. However, unlike superoxide, H_2_O_2_ can rapidly diffuse across cell membranes, and in the presence of transition metal ions, it can be converted to hydroxyl radicals via Fenton chemistry [44]. Highly reactive hydroxyl radicals are then formed in the presence of Fe_2_^+^ and H_2_O_2_ according to the Fenton reaction. The imbalance ratio of the antioxidant enzymes and oxidants (ROS) results in the emergence of illness [45]. Glutathione peroxidases present in cytosol and mitochondria have a major role in converting hydroxyl radical (OH^−^) into water. In this mechanism, hydroxyl production is affected [46]. Our results assessed that the reduced levels of GSH were restored to normal when diabetic animals were treated with 15 mg/kg of pancreatic 1,3,4-oxadiazole derivative. In liver, 10 and 15 mg/kg of 1,3,4-oxadiazole derivative showed a significant (*P* < 0.05) increase in the GSH level. Similarly, 10 and 15 mg/kg of 1,3,4-oxadiazole derivative showed a significant (*P* < 0.01) increase in the GSH level in the kidney as compared to the diabetic group.

The current data revealed that consistent hyperglycemia via alloxan-generated ROS caused marked oxidizing effects, as evidenced by an increase in diabetic animal MDA rates compared to the nondiabetic animals [47]. High levels of MDA were reduced to normal values by 1,3,4-oxadiazole derivative treatment. The 1,3,4-oxadiazole derivative at a dose of 15 mg/kg showed a highly significant reduction in the MDA serum level in the liver (*P* < 0.001) and kidney (*P* < 0.01), whereas nonsignificant reduction was reported in the pancreas as compared to the diabetic group. The protein level was increased in 1,3,4-oxadiazole derivative as compared to the diabetic groups. MDA is the mutagenic in bacteria and mammalian cells [48].

Histology of the pancreas demonstrates normal acini and normal cellularity in the control islets of Langerhans. In diabetic animals, extensive damage to Langerhans islets was treated and reduced islet dimensions were observed too. On the other hand, at a dose level of 15 mg/kg, 1,3,4-oxadiazole derivative showed hypertrophy and vacuolations of Langerhans *β* cells.

In the serum of diabetic rats, hepatic enzymes have increased [49]. This may mainly be due to the leakage of these enzymes from the liver cytosol to the bloodstream as a result of the hepatotoxic alloxan effect. 1,3,4-oxadiazole derivative reduced the serum ALT and AST levels at all doses that showed a protective effect and normal liver function in restoring impairment to organs due to diabetes.

Cholesterol and triglyceride play a major role in the pathogenesis of DM-related complications. The unusually high serum cholesterol and triglyceride content in diabetics were primarily due to the increased production of free fatty acids from peripheral fat depots. As lipase is suppressed by insulin, insulin insufficiency or resistance may result in dyslipidemia [50]. The group treated with 1,3,4-oxadiazole derivative showed changes in the cholesterol and triglyceride profile in contrast to the diseased one.

Diabetic hyperglycemia contributes to an increase in urea and creatinine plasma levels, which are regarded as important markers of renal dysfunction [51]. With respect to the control group, the findings showed an increase in plasma levels of urea and creatinine in the diseased group. These findings suggested that diabetes may cause renal dysfunction. When compared to the diseased group, treatment with an 1,3,4-oxadiazole derivative significantly (*P* < 0.001) decreased the plasma urea level. Similarly, the level of creatinine was decreased (*P* < 0.001) after the administration of 1,3,4-oxadiazole derivative in comparison to the diseased group.

Hepatic photomicrographs displayed the normal liver cells through a well-preserved cytoplasm, nucleus, core nuclei, and main vein in the normal group. The standard lobular structure was conserved in diabetic rats. The main vein detrimentally clogged up. Focal hemorrhage was also established. The accumulation of fat was noticeable. Kupfer cell activation and congestion of the central vein can be seen in the liver treated with 15 mg/kg of 1,3,4-oxadiazole derivative. In normal animals, the histology of the glomeruli and the Bowman capsule is appropriate. For diabetic rats, the basement membrane of the arterioles of glomeruli is slightly thickened, including a slight change in mesangial mesangium density. Epithelial proteins were deposited in the lumen of the renal tubules after 15 mg/kg of oxadiazole derivative treatment.

The potential mechanism for oxadiazole derivative action was found to be a high-affinity ligand of the receptor-activated peroxisome proliferator, a member of the superfamily of nuclear receptors that cause transcription of insulin-responsive genes [52]. The glucose-lowering effect of oxadiazole derivative is mediated through the improvement of insulin sensitivity on the cell membrane, causing an increased uptake of glucose into the cell for energy [40]. In addition, oxadiazole derivative caused regranulation of pancreatic beta cells in pancreatectomised rats [53].

Numerous studies have been conducted on Nrf2 as a potential antioxidant signaling mechanism [54]. Nrf2 acts as an inactive dimer due to its cytoplasmic association with the Kelch-like ECH-associated protein 1. Nrf2 activation is attenuated by Keap1, and its nuclear translocation is prevented. The complex splits whenever a stress signal is detected, permitting Nrf2 to move freely into the nucleus and activates the translocation, where it triggers the antioxidant enzymes. Nrf2 binds to antioxidant response elements in the nucleus and promotes the transcription of endogenous defensive enzymes [55]. As the previous results shows that the quantity of antioxidant enzymes was increased by treating the rat with oxadiazole derivative, so the mechanistic studies showed that 1,3,4-oxadiazole derivative could trigger Nrf2 nuclear translocation, subsequently resulting in increased expression of Nrf2 target gene. Meanwhile, oxadiazole derivative suppressed the increase of the ROS level [56].

Various groups of heterocyclic and fused heterocyclic molecules have been described in search of antiobesity, antidiabetes, anti-inflammatory, antibiotic, and anticancer agents through scientific analysis and drug design approach [57]. Oxadiazole occurs in four feasible interpretations of isomers, but, 1,3,4-oxadiazole is broadly researched for different functions [58]. 1,3,4-heterocycles of oxadiazole have different pharmacological behaviors, including antioxidative traits [36]. It also possesses anticancer activity [59]. 1,3,4-oxadiazole shows antidiabetic activity by inhibiting the glycogen phosphorylase enzyme [60]. Large numbers of clinically used medicinal compounds have oxadiazole as a pharmacophore, e.g., raltagravir, furamizole, zibotentane, tiodazosin, and nesapidil based on 1,3,4-oxadiazole moiety [61]. An oxadiazole ring is included in peroxisome proliferator-activated receptors (PPAR), one of the most well-known antidiabetic prescribed medicines in the market [62–68]. Due to the increased resistance of oxadiazole derivatives, a novel oxadiazole derivative with increased activity as therapeutics needs to be introduced in the market.

## 5. Conclusions

It can be concluded that the imbalance between oxidants and antioxidant enzymes play a pivotal role in development and progression of diabetes. In this study, the 1,3,4-oxadiazole derivative produced pronounced antidiabetic activity at a dose of 15 mg/kg, showing substantial decrease in the blood glucose level, body weight, and Hb1Ac level. Other biochemical metabolic parameters including ALT, AST, urea, creatinine, triglyceride, and cholesterol were also reduced by 1,3,4-oxadiazole derivative. The antioxidant enzyme activity was significantly increased by 1,3,4-oxadiazole derivative. The exact mechanism by which 1,3,4-oxadiazole derivative decreases the level of blood glucose in diabetic rats requires further analysis. Moreover, further studies are needed to explore the pharmacological profile of 1,3,4-oxadiazole derivative.

## Figures and Tables

**Table 1:** Estimation of Hb1Ac and insulin levels.

Treatment groups	Hb1Ac (% of total Hb)	Insulin (*μ*U/ml)
Normal control	0.375 ± 0.01	7.350 ± 0.05
Disease control	0.735 ± 0.005	3.850 ± 0.05
Glimepiride	0.425 ± 0.015^*∗∗*^	4.250 ± 0.15
Acarbose	0.340 ± 0.040^*∗∗*^	3.950 ± 0.05^*∗∗*^
OXD mg/kg	0.565 ± 0.005	3.850 ± 0.05
OXD 10 mg/kg	0.445 ± 0.005^*∗*^	4.250 ± 0.15^*∗∗*^
OXD 15 mg/kg	0.395 ± 0.005^*∗∗*^	5.650 ± 0.05^*∗∗∗*^

Data are presented as mean ± SEM, *n* = 6, ^*∗∗∗*^*P* < 0.001, ^*∗∗*^*P* < 0.01, and ^*∗*^*P* < 0.05 as compared with the disease group. *Note.* OXD: 1,3,4-oxadiazole derivative.

**Table 2:** Effect of 1,3,4-oxadiazole derivative on biochemical parameters in pancreas tissue.

Treatment groups	SOD (*μ*g/mg of protein)	CAT (*μ*gmole/min/mg of protein)	GSH (*μ*g/mg of protein)	MDA (*μ*mole/mg of protein)	Protein (*μ*g/mg of protein)
Normal control	43.5 ± 0.01	150 ± 0.5	25.5 ± 0.5	0.165 ± 0.005	460 ± 0.5
Disease	35.5 ± 0.5	96 ± 0.5	20.5 ± 0.5	0.390 ± 0.010	390 ± 0.5
Glimepiride	44.5 ± 0.5^*∗∗*^	122 ± 1.5^*∗*^	24.5 ± 0.5	0.375 ± 0.005	627 ± 1.0^*∗∗*^
Acarbose	55.5 ± 0.5^*∗∗∗*^	125 ± 0.5^*∗*^	26.5 ± 0.5^*∗*^	0.365 ± 0.005	656 ± 0.5^*∗∗*^
OXD 5 mg/kg	41.5 ± 0.5^*∗*^	120 ± 0.5^*∗*^	23.5 ± 0.5	0.380 ± 0.010	480 ± 0.5^*∗*^
OXD 10 mg/kg	43.5 ± 0.5^*∗∗*^	121 ± 0.5^*∗*^	24.5 ± 0.5	0.375 ± 0.005	626 ± 0.5^*∗∗*^
OXD 15 mg/kg	53.5 ± 0.5^*∗∗∗*^	123 ± 1.0^*∗*^	25.5 ± 0.5^*∗*^	0.370 ± 0.005	629 ± 1.0^*∗∗*^

Data are presented as mean ± SEM, *n* = 6, ^*∗∗∗*^*P* < 0.001, ^*∗∗*^*P* < 0.01, and ^*∗*^*P* < 0.05 when compared with alloxan-treated groups. *Note.* OXD: 1,3,4, oxadiazole derivative.

**Table 3:** Antioxidant activity of 1,3,4-oxadiazole derivative in liver and kidney tissue.

Treatment groups		SOD (*µ*g/mg of protein)	CAT (*µ*gmole/min/mg of protein)	GSH (*µ*g/mg of protein)	MDA (*µ*mole/mg of protein)	Protein (*µ*g/mg of protein)
*Liver*						
Normal control		37.73 ± 0.50	0.886 ± 0.009	33.72 ± 0.01	0.835 ± 0.001	881 ± 0.1
Disease		5.590 ± 2.01	0.450 ± 0.005	9.830 ± 0.01	1.300 ± 0.009	341 ± 0.1
Glimepiride		11.155 ± 0.005^*∗*^	0.860 ± 0.004^*∗∗*^	16.350 ± 0.01^*∗*^	0.942 ± 0.002^*∗∗*^	451 ± 0.1^*∗*^
Acarbose		11.170 ± 0.010^*∗*^	0.839 ± 0.039^*∗∗*^	16.365 ± 0.02^*∗*^	0.933 ± 0.001^*∗∗*^	452 ± 0.5^*∗*^
OXD 5 mg/kg		7.51 ± 0.009	0.780 ± 0.005^*∗*^	10.865 ± 0.014	1.120 ± 0.009^*∗*^	391 ± 0.1
OXD 10 mg/kg		11.11 ± 0.005^*∗*^	0.822 ± 0.005^*∗∗*^	15.900 ± 0.009^*∗*^	0.966 ± 0.001^*∗∗*^	411 ± 0.1
OXD 15 mg/kg		11.175 ± 0.005^*∗*^	0.891 ± 0.005^*∗∗*^	16.370 ± 0.009^*∗*^	0.9015 ± 0.002^*∗∗*^	454 ± 0.5^*∗*^

*Kidney*						
Normal control	68.75 ± 0.55		0.985 ± 0.004	18.34 ± 0.50	0.947 ± 0.043	196 ± 0.5
Disease	44.05 ± 0.54		0.445 ± 0.005	4.57 ± 1.4	1.760 ± 0.049	166 ± 0.5
Glimepiride	49.05 ± 0.10^*∗∗*^		1.110 ± 0.009^*∗*^	12.20 ± 0.50^*∗∗*^	1.030 ± 0.080^*∗∗*^	174 ± 1.0^*∗∗*^
Acarbose	54.05 ± 0.55^∗∗∗^		1.135 ± 0.004^*∗*^	12.43 ± 0.004^*∗∗*^	1.225 ± 0.004^*∗*^	183 ± 0.5^*∗∗∗*^
OXD 5 mg/kg	48.05 ± 0.54^*∗*^		0.925 ± 0.004	9.54 ± 0.46^*∗*^	1.010 ± 0.005^*∗∗*^	168 ± 0.5
OXD 10 mg/kg	48.75 ± 0.54^*∗∗*^		1.090 ± 0.009^*∗*^	11.62 ± 0.50^*∗∗*^	1.005 ± 0.095^*∗∗*^	170 ± 0.5
OXD 15 mg/kg	48.90 ± 0.10^*∗∗*^		1.100 ± 0.009^*∗*^	12.12 ± 0.52^*∗∗*^	0.891 ± 0.090^*∗∗∗*^	172 ± 1.0^*∗*^

Data are presented as mean ± SEM (*n* = 6). ^*∗∗∗*^*P* < 0.001, ^*∗∗*^*P* < 0.01, and ^*∗*^*P* < 0.05 represent significantly different values as compared to the disease group. *Note.* OXD: 1,3,4-oxadiazole derivative.

## Data Availability

The data used to support the findings of this study are included within the article.
